# Short-Term Facility-Based Functional Electrical Stimulation for Chronic Post-Stroke Foot Drop: A Pilot Study

**DOI:** 10.3390/bioengineering13020238

**Published:** 2026-02-18

**Authors:** Diana-Lidia Tache-Codreanu, Ioana Angela Rotaru, Mihai-Andrei Butum-Cristea, Georgeta Stefan, Andrei Tache-Codreanu, Corina Sporea, Ana-Maria Tache-Codreanu

**Affiliations:** 1 Medical Rehabilitation Department, Colentina Clinical Hospital, Stefan cel Mare Street No. 19–21, 020125 Bucharest, Romania; diana.tache@spitalul.colentina.ro (D.-L.T.-C.); angela.rrotaru@yahoo.com (I.A.R.); mihai5020@gmail.com (M.-A.B.-C.); 2 Faculty of Veterinary Medicine, University of Agronomic Sciences and Veterinary Medicine, 105 Splaiul Independentei Street, 050097 Bucharest, Romania; 3 Faculty of Theatre, The National University of Theatre and Film “I.L. Caragiale”, 75–77 Matei Voievod Street, 021452 Bucharest, Romania; 4 Faculty of Midwifery and Nursing, University of Medicine and Pharmacy “Carol Davila”, 37 Dionisie Lupu Street, 020021 Bucharest, Romania; corina.sporea@gmail.com; 5 National Teaching Center for Children’s Neurorehabilitation “Robanescu-Padure”, 44 Dumitru Minca Street, 041408 Bucharest, Romania; 6 Faculty of Medicine and Farmacy, University of Medicine and Pharmacy “Carol Davila”, 37 Dionisie Lupu Street, 020021 Bucharest, Romania; ana-maria.tache2024@stud.umfcd.ro

**Keywords:** stroke, gait rehabilitation, neurorehabilitation, lower limb motor function, functional electrical stimulation

## Abstract

Background: Functional Electrical Stimulation (FES) for post-stroke drop foot is commonly applied in acute and subacute stroke rehabilitation or as part of long-term home-based programs in chronic patients. Evidence supporting short facility-based rehabilitation programs incorporating FES in chronic populations remains limited. The aim of this study was to explore functional outcomes associated with such a program in a chronic population. Materials and methods: A 10-day facility-based rehabilitation program incorporating FES therapy followed by 3-month follow-up was delivered to 14 chronic post-stroke patients with foot drop (8 women; aged 62.6 ± 12.2 years). FES was applied during walking with stimulation synchronized to the swing phase of gait (35 Hz, 300 μs, 15 min per session). Activities of daily living and mobility were assessed using clinical outcome measures. Statistical significance (*p* < 0.05), effect sizes, and minimal clinically important difference (MCID) responder rates were evaluated. Results: Statistically significant improvements were observed across all outcome measures post-treatment and at follow-up, with MCID responder rates exceeding 50%. Conclusions: A short facility-based multimodal rehabilitation program incorporating FES was associated with functional improvements in chronic post-stroke patients. Given the multimodal design, these findings cannot be attributed to FES alone and should be interpreted as exploratory.

## 1. Introduction

Stroke is a leading cause of serious long-term disability and remains a major global health burden, with over 12.2 million new strokes occurring each year worldwide [1]. Persistent motor impairments limit upper limb mobility in up to 40–50% of post-stroke survivors and walking limitations remain in about 50% of patients even six months after starting rehabilitation [2,3,4].

Foot drop, insufficient ankle dorsiflexion during swing, often accompanied by inappropriate plantarflexor activity, affects an estimated 20–30% of stroke survivors [5]. Impaired toe clearance and reduced ankle control contribute to compensatory strategies at proximal joints, slower and more asymmetric gait, and an increased risk of falls [6,7,8]. This can significantly affect a patient’s independence in activities of daily living and community reintegration [9]. Up to 95% of stroke survivors require some degree of assistance in performing activities of daily living, and approximately 90% experience unrecognized depression [10]. Stroke also has a documented impact on psychosocial well-being—up to 44% of stroke survivors report feelings of loneliness [11]. Comparable limitations in functional independence and daily activity performance have also been reported in patients undergoing structured rehabilitation programs for other chronic neurological conditions in real-world clinical settings [12,13].

Conventional management of post-stroke foot drop typically relies on the use of ankle-foot orthoses (AFOs), which can effectively improve gait safety and reduce compensatory movements. However, AFOs act as a passive mechanical support and do not address the underlying neuromuscular deficits. Because active muscle contraction is not facilitated, this approach may contribute to restricted ankle mobility, muscle atrophy, and long-term weakness [14,15,16]. In contrast, Functional Electrical Stimulation (FES) delivers timed, task-specific activation of the peroneal nerve and dorsiflexor muscles during the swing phase of gait. This activity-dependent stimulation promotes more physiological movement patterns and may enhance motor relearning and neuroplasticity through repeated use-dependent activation [14,17,18,19]. Moreover, clinical evidence has demonstrated not only the immediate orthotic benefits of FES while worn, but also a therapeutic (training) effect resulting from repeated use, which can persist even after the device is removed. This carryover effect is attributed to motor relearning and neuroplastic adaptations induced by task-specific, activity-dependent stimulation [14,20].

One of the limitations of FES is the relatively high interindividual variability in treatment response, which may depend on the extent of neurological impairment, body composition, electrode placement, and stimulation parameters. This variability highlights the need for quantitative and predictive approaches that go beyond empirical parameter tuning. Recently published work has demonstrated the potential of hybrid frameworks combining finite element modeling and artificial intelligence (AI) to capture complex interactions within biomedical systems using subject-specific and device-related data [21]. Although not applied directly in the context of neuromuscular stimulation, such approaches may, in the future, support individualized prediction and optimization of FES-based interventions.

At the neurophysiological level, experimental studies have provided important insights into the mechanisms underlying activity-dependent effects of functional electrical stimulation. In animal models, adaptive behavioral changes following FES have been shown to be mediated by neural circuits at different levels of the nervous system. Hook and Grau demonstrated that functional improvements following electrical stimulation in rats with thoracic spinal cord injury were primarily mediated by spinal neural circuits, highlighting the capacity of activity-dependent stimulation to modulate intrinsic motor networks [22]. Extending these observations, Ceccato et al. investigated the effects of repeated FES in rats with experimentally induced ischemic cortical lesions and reported improved motor performance in FES-treated animals compared with controls. Histological analyses further revealed increased density of MAP-immunoreactive fibers in peri-lesional cortical regions, suggesting structural changes associated with FES-mediated functional recovery [23]. Together, these experimental findings support the concept that activity-dependent electrical stimulation can influence neural network organization under controlled experimental conditions.

Although the clinical evidence on FES is relatively rich regarding its immediate orthotic effects during device use and short-term therapeutic effects immediately following long-term, predominantly home-based training programs, studies that systematically evaluate the persistence of the therapeutic, so-called carryover effect several months after completion of an ambulatory program are virtually absent [14,18,19,20]. Consequently, it remains unclear whether walking ability and independence in activities of daily living deteriorate remain stable or continue to improve as a result of ongoing neuroplastic processes triggered by FES. This question is particularly relevant in the chronic phase after stroke, where spontaneous neurological recovery is limited and functional changes following short-term interventions are generally expected to be modest compared with those observed in acute and subacute stages [14].

The primary aim of this study is to evaluate the effect of a relatively short facility-based multimodal rehabilitation program incorporating FES in individuals with chronic stroke (≥6 months post-onset) on functional mobility and independence in activities of daily living. The study will assess the magnitude of functional improvement immediately after completion of the program and, importantly, its persistence or change following a 3-month follow-up period.

## 2. Materials and Methods

### 2.1. Study Design

This retrospective single-arm pilot study was conducted as part of the standard rehabilitation program between March and December 2025 at the Rehabilitation Department in Colentina Clinical Hospital, Bucharest, Romania.

All data were collected during routine clinical care and were analyzed retrospectively. No prospective screening or recruitment process was performed, and patients were retrospectively identified from clinical records based on eligibility criteria. The study cohort therefore represents a subset of patients who completed the full rehabilitation program were available for analysis.

All participants provided written informed consent for treatment and for the use of anonymized clinical data for research and publication purposes.

The study protocol was approved by the Research Ethics Committee of Colentina Hospital, Bucharest on 3 November 2025, approval number 38, and was conducted in accordance with the ethical principles of the Declaration of Helsinki.

### 2.2. Participants

The study included adult patients in the chronic phase after stroke (≥6 months post-onset) and clinically evident foot drop who were able to ambulate at least 10 m with or without the use of an assistive device such as a cane or walker.

Eligible participants were required to have a stable medical and psychological condition, without acute decompensation of any chronic disease, and to demonstrate adequate ankle stability during the stance phase of gait. Additional inclusion criteria included no anticipated medication changes for at least six months and no prior experience with FES for foot drop.

Patients were excluded if they had a history of cardiovascular disorders (such as myocardial infarction, congestive heart failure, or the presence of a permanent pacemaker), seizure disorders, severe lower-limb pathologies, or morbid obesity (body mass index > 40 kg/m^2^), all of which could interfere with or reduce responsiveness to FES. Further exclusion criteria included a baseline gait speed exceeding 1.2 m/s, a documented history of recurrent falls before the stroke event, or clinical evidence of lower motor neuron lesions or peripheral neuropathies that could result in an inadequate motor response to stimulation.

During the study period, a total of 27 patients with post-stroke foot drop underwent the facility-based rehabilitation program incorporating FES. Of these, 10 patients did not meet the eligibility criteria due to being in the acute or subacute stage after stroke, and 3 patients did not complete the full intervention period. The final analysis therefore included 14 patients (8 women and 6 men) with a mean age of 62.6 ± 12.2 years and a mean body mass index of 26.8 ± 3.7 kg/m^2^ who fulfilled all eligibility criteria and completed the full treatment program. The mean time since stroke onset was 2.1 ± 1.1 years. All participants tolerated the intervention well, and no adverse events were reported.

### 2.3. Intervention

All patients participated in a 10-day comprehensive rehabilitation program focused on improving gait function and lower-limb motor control after stroke. The program was delivered as a multimodal, facility-based rehabilitation intervention as part of routine clinical care and included a combination of standard therapies for post-stroke hemiparesis, namely electrotherapy, kinesiotherapy, and therapeutic massage, performed daily under the supervision of a physiotherapist.

Electrotherapy was delivered using longitudinal galvanic stimulation applied to the affected lower limb to facilitate nerve and muscle excitability. Therapeutic ultrasound was applied to the paravertebral lumbar region to induce reflex effects on the lower limbs. Therapeutic massage using tonic techniques was performed to stimulate local blood circulation and enhance nutrient delivery, with the aim of supporting muscle strength and preventing muscle atrophy.

Kinesiotherapy consisted of exercises focused on functional mobility, including bed mobility and transfers (from supine to sitting and from sitting to standing), passive, active-assisted, and active mobilization exercises, gait training with or without an assistive device (cane), as well as balance and coordination exercises performed in standing.

**Figure 1:**
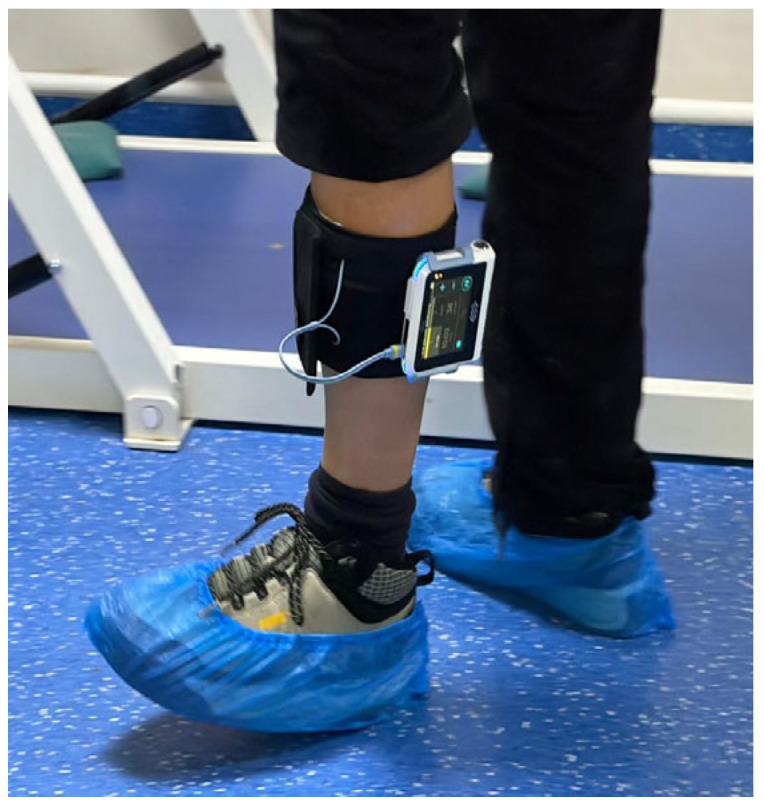
Setup of the rehabilitation intervention during the study course.

In addition to the standard rehabilitation regimen, patients received FES (BTL Walk, BTL Industries Ltd., Prague, Czech Republic) targeting the peroneal nerve and dorsiflexor muscles of the affected limb. FES was administered using a stimulator delivering rectangular, biphasic impulses with equal intensity and duration phases, a frequency of 35 Hz, and a pulse duration of 300 µs. FES was applied as one component of the multimodal rehabilitation program, synchronized to the swing phase of gait to facilitate dorsiflexor activation during walking. Stimulation was applied through disposable self-adhesive electrodes: the active (negative) electrode was placed over the motor point of the tibialis anterior muscle, while the indifferent (positive) electrode was positioned approximately 5–7 cm proximally along the peroneal nerve trajectory (Figure 1). Each FES session was performed during 15 min of walking on flat ground. The FES intervention was delivered once daily throughout the 10-day program in combination with other rehabilitation modalities, as an integral component of the individualized rehabilitation treatment. During the subsequent 3-month follow-up period, patients were advised to continue with kinesiotherapy as per routine clinical recommendations.

### 2.4. Outcome Measures

Functional outcomes routinely assessed as part of standard clinical care were extracted retrospectively from medical records and were available at baseline (T0), immediately after completion of the rehabilitation program (T1), and at approximately 3 months after discharge (T2).

Functional mobility was evaluated at all time points, while independence in activities of daily living, assessed using the Barthel Index and the Lawton Instrumental Activities of Daily Living scale (IADL), was evaluated at T0 and T2 only. After the 3-month follow-up assessment, some patients were contacted by telephone as part of routine clinical practice; these contacts were not standardized and were not included in the formal analysis.

To determine whether observed changes represented meaningful functional improvement, all measures were interpreted relative to their Minimal Clinically Important Difference (MCID) using published thresholds applicable to post-stroke populations when available.

The Barthel Index is a widely used clinical instrument for assessing a patient’s ability to perform basic activities of daily living. It evaluates ten key functions, including feeding, bathing, dressing, bowel and bladder control, mobility, and stair use. Each activity is rated according to the level of assistance required, resulting in a total score ranging from 0 to 100, where higher scores indicate greater functional independence [24]. Scores between 0 and 20 reflect total dependency, 21 to 60 indicate severe dependency, 61 to 90 correspond to moderate dependency, 91 to 99 represent slight dependency, and a score of 100 indicates full independence [25]. The Barthel Index is quick to administer, typically requiring only 5 to 10 min, and is simple to score while maintaining high reliability across various clinical settings. It is routinely used in stroke rehabilitation, geriatric care, neurological and physical therapy, and discharge planning to quantify functional independence and monitor recovery over time [24]. Recent evidence suggests that a change of approximately 10 points on the 100-point Barthel Index scale represents a MCID for post-stroke populations [26]. Despite its practicality and strong evidence base, the Barthel Index has several limitations. It may exhibit a ceiling effect in high-functioning patients and does not assess cognitive, emotional, or social aspects of independence. Nevertheless, it remains a gold standard for evaluating functional recovery and independence due to its simplicity, responsiveness, and extensive validation in stroke rehabilitation research.

The Lawton IADL scale was used to assess eight instrumental daily functions (ability to use the telephone, shopping, food preparation, housekeeping, laundry, mode of transportation, responsibility for own medication, and ability to handle finances), with a total score ranging from 0 to 8, where higher scores indicate greater independence. As no specific MCID has been established for the Lawton IADL scale in post-stroke populations, a ≥1-point increase from baseline was pragmatically adopted as the threshold for clinically meaningful functional improvement, in line with previous geriatric and rehabilitation studies, reflecting increased independence in at least one instrumental activity [27].

The 10-Meter Walk Test (10MWT) is a simple, reliable, and widely used clinical assessment tool designed to measure an individual’s walking speed over a short distance. It is commonly used in rehabilitation settings to monitor progress in patients with neurological conditions such as stroke, spinal cord injury, or Parkinson’s disease, as well as in those with orthopedic impairments or general mobility limitations. During the test, the individual is instructed to walk a distance of 10 m at a comfortable or maximum safe speed. In the present study, the test was performed once at each assessment time point, and participants were instructed to walk at their comfortable, self-selected walking speed to minimize fall risk.

To ensure accuracy, only the middle six meters are timed, allowing the first and last two meters for acceleration and deceleration. Walking speed is calculated by dividing the timed distance (6 m) by the time taken to walk it, and the result is expressed in meters per second (m/s) [28]. Results of the 10MWT are typically interpreted according to the following functional categories: individuals walking at speeds below 0.4 m/s are classified as household ambulators, those between 0.4 and 0.8 m/s as limited community ambulators, speeds between 0.8 and 1.2 m/s correspond to community ambulators, and speeds above 1.2 m/s indicate full community ambulation [29]. Recent evidence by Hosoi and Kamimoto suggests that the MCID in post-stroke populations may vary according to baseline gait speed, with thresholds of 0.05 m/s for slow walkers (<0.4 m/s), 0.11 m/s for moderate walkers (0.4–0.8 m/s), and 0.21 m/s for fast walkers (>0.8 m/s), providing a more individualized interpretation of clinically relevant change [30].

The Timed Up and Go (TUG) test is a simple, quick, and reliable clinical assessment used to evaluate an individual’s overall mobility, dynamic balance, and functional ability. It is widely applied in rehabilitation and geriatric settings, particularly for identifying fall risk and monitoring changes in mobility among individuals with neurological or musculoskeletal conditions. During the test, the individual is instructed to stand up from a standard armchair, walk a distance of approximately three meters, turn around, walk back to the chair, and sit down.

In this study, the TUG was performed once at each assessment time point, with participants instructed to complete the task at a comfortable, self-selected pace to ensure safety. The total time required to complete this sequence is recorded, with shorter times reflecting better mobility and balance. For healthy adults, completion times under ten seconds are considered normal, while times exceeding 12 to 15 s are typically associated with impaired mobility and increased fall risk [31,32]. In stroke rehabilitation, the TUG has demonstrated strong reliability and responsiveness, with an improvement of approximately 2.9 s identified as the MCID representing a meaningful functional gain [33].

For all outcomes, changes equal to or greater than the established or pragmatically defined MCID values were interpreted as clinically meaningful improvements in functional performance.

### 2.5. Statistical Analysis

For statistical analysis and data processing, IBM SPSS Statistics, version 26 (IBM Corp., Chicago, IL, USA) and Microsoft Excel 2021, Office Professional Plus (Microsoft Corp., Redmond, WA, USA) were used.

The final dataset included fourteen participants, consistent with the pilot and exploratory nature of the study. The analysis was primarily intended to assess the feasibility of the short-term facility-based FES rehabilitation program and to identify trends in the outcomes observed during the follow-up period, rather than to draw generalizable conclusions or demonstrate the long-term efficacy of the intervention.

Data were first tested for normality using the Shapiro–Wilk test. Given the pilot nature of the study and the small sample size, data are presented as mean ± standard deviation (SD) as well as median and interquartile range (IQR). The Wilcoxon signed-rank test was used to assess the statistical significance of changes as the hypothesis of normal distribution was rejected. Effect sizes were calculated as r, defined as the standardized test statistic (Z) divided by the square root of the number of paired observations (r = Z/√N). Values of 0.1, 0.3, and 0.5 were interpreted as small, medium, and large effects, respectively [34]. Statistical significance was set at *p* < 0.05. No a priori power calculation was performed due to the retrospective and exploratory design. Effect sizes were reported to estimate the magnitude of observed changes and inform future study design.

In addition to statistical significance, clinical significance was evaluated using the MCID thresholds established for each outcome measure. The MCID responder rate was then calculated as the percentage of participants who achieved or exceeded the MCID for each parameter. Due to the risk of ceiling effects for the Barthel Index and the IADL scale, participants with baseline scores that did not allow attainment of the MCID (defined as ≥95 points for the Barthel Index and 8 points for the IADL) were excluded from the MCID responder rate analysis.

## 3. Results

An overview of questionnaire scores and performance-based measurements obtained throughout the study course is presented in Table 1. The results of the statistical analyses are summarized in Table 2. Compared with baseline values, statistically significant improvements with large effect sizes were observed across all outcome measures both post-treatment and at follow-up. These findings were further supported by MCID responder rates exceeding 50%, indicating clinically meaningful improvements.

**Figure 2:**
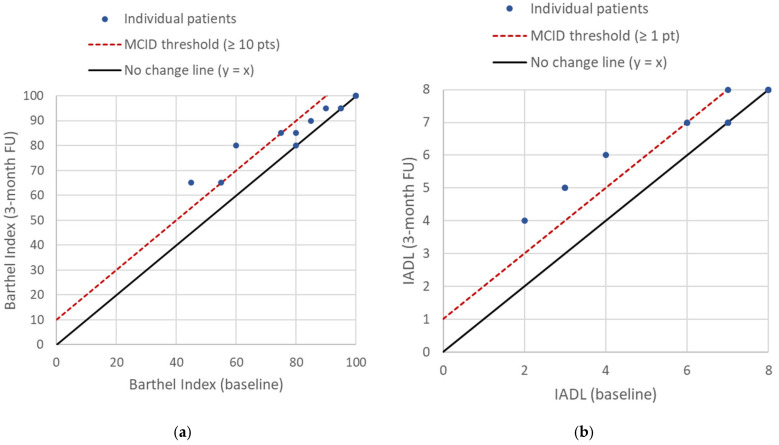
Visualization of patient-level changes in (**a**) the Barthel Index and (**b**) the IADL score between baseline and the 3-month follow-up. Each dot represents an individual patient. Dots located on the black line indicate no change, dots between the black and red lines represent improvement below the MCID threshold, and dots on or above the red line indicate clinically meaningful improvement according to the MCID.

Graphical representations of patient progression in terms of independence in daily activities are shown in Figure 2. The figure illustrates that no patient experienced deterioration over the course of the study. In some participants, achievement of the MCID was not possible due to ceiling effects associated with high baseline scores.

**Figure 3:**
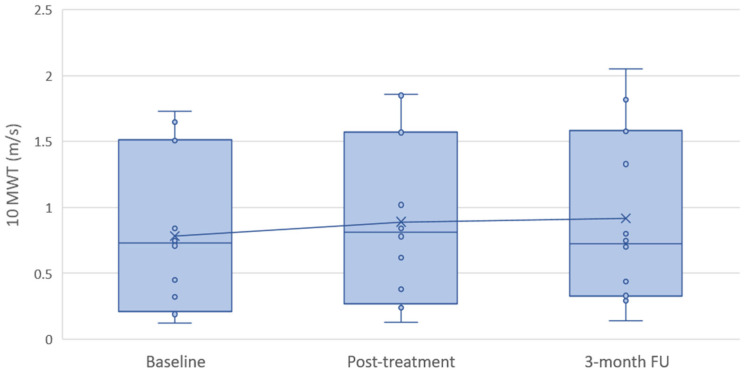
Distribution of 10MWT walking speed at baseline, post-treatment, and 3-month follow-up. Boxplots represent the median and interquartile range, with whiskers indicating the data range. Individual dots represent individual participants, and the cross indicates the group mean at each time point. The connecting line illustrates the change in group mean walking speed over time.

**Figure 4:**
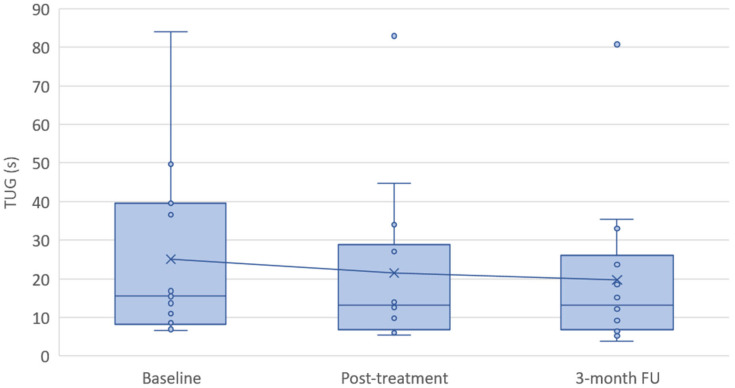
Distribution of TUG time at baseline, post-treatment, and 3-month follow-up. Boxplots represent the median and interquartile range, with whiskers indicating the data range. Individual dots represent individual participants, and the cross indicates the group mean at each time point. The connecting line illustrates the change in group mean TUG performance over time.

Data from the performance-based mobility measures are presented graphically in Figure 3 and Figure 4 for 10MWT walking speed and TUG time, respectively. During the 3-month follow-up period, a decline in functional test performance compared with baseline values was observed in a small number of participants. This was evident in three participants for 10MWT walking speed and in two participants for TUG performance. The remaining participants demonstrated a predominantly improving trend, which was largely maintained after completion of the active intervention.

## 4. Discussion

The present study suggests that even a short multimodal rehabilitation program incorporating FES may be associated with functional improvements in patients in the chronic phase after stroke, with changes observed during the intervention period and maintained at follow-up. The observed improvements should be interpreted in the context of a rehabilitation program combining multiple therapeutic modalities. Based on the design of the present study, it is not possible to attribute the observed effects to FES as an isolated intervention.

Given the small sample size, the observed statistical significance should be interpreted with caution. Although large effect sizes were observed across all outcome measures, these estimates may be inflated in small samples and in the context of multiple comparisons and should therefore be interpreted cautiously.

Clinical relevance was supported by MCID responder rates exceeding 50% in all parameters. MCID thresholds were selected based on existing evidence, and considering the short intervention protocol, the chronic status of the participants, and the pilot nature of the study, the observed outcomes may be regarded as clinically meaningful.

Although no deterioration was observed in any participant with respect to activities of daily living, as assessed by the Barthel Index and IADL, some participants demonstrated a decline in functional mobility outcomes (10MWT and TUG) during the follow-up period, in some cases even compared with baseline values. This apparent inconsistency may be explained by differences in test sensitivity, as well as by the subjective perception of how gait quality influences daily functioning. In addition, in two participants, the decline in mobility performance temporally coincided with the onset or exacerbation of musculoskeletal conditions, such as knee osteoarthritis, which may have contributed to reduced walking performance during follow-up. A modest reduction in walking speed may not necessarily translate into a perceived limitation in daily activities, and vice versa. Moreover, despite clinically meaningful improvements in 10MWT and TUG performance exceeding the MCID in some participants, these changes were not consistently reflected in measures of activities of daily living, which remained unchanged.

Chronicity of the post-stroke condition is often considered a limiting factor for functional improvement, primarily due to reduced cerebral plasticity compared with the acute phase [14,35]. Most rehabilitation programs incorporating FES in hospital or rehabilitation center settings predominantly target patients in the acute or subacute phase after stroke and typically involve a substantially higher treatment intensity than that applied in the present study. Choi et al. investigated a cohort of 18 patients with a mean time since stroke of approximately 18 weeks, corresponding to the subacute phase, and applied a protocol consisting of 20 treatment sessions. Improvements in walking speed assessed by the 10MWT were comparable to those observed in the present study [36]. However, differences in patient populations, intervention composition, and study design limit direct comparability. To date, benefits associated with FES therapy in chronic post-stroke patients have been reported mainly in the form of case reports following long-term home-based programs, which generally exceed the intensity feasible within facility-based rehabilitation. For example, David et al. reported functional improvements in a 22-year chronic post-stroke patient following a six-month home-based FES intervention [14].

Within the multimodal rehabilitation program, FES may have contributed to the observed functional improvements through its mechanisms of action. Dynamic, task-specific stimulation enabling active engagement of the dorsiflexor muscles may promote a more physiological gait pattern and improved timing during the swing phase of gait [37]. Through sensorimotor facilitation, FES provides enhanced afferent input that may support motor relearning and, to some extent, neural plasticity [37,38]. It can be hypothesized that a sufficiently intensive rehabilitation program may lead to more durable functional adaptations that persist even after discontinuation of FES therapy [39]. In addition, patients may experience increased confidence and balance during walking, accompanied by a reduced fear of falling [39,40]. Beyond motor performance, structured physical rehabilitation programs have also been associated with improvements in emotional well-being, including reductions in anxiety and depressive symptoms, even when the primary therapeutic focus is physical recovery [41,42,43]. Such psychosocial benefits may indirectly support greater participation in daily activities and sustained engagement in mobility-related tasks. These combined effects of a multimodal rehabilitation approach may therefore better explain the observed improvements than neural plasticity in the sense of cortical reorganization, particularly given the short duration of the intervention and the chronic status of the studied population.

Although substantial spontaneous recovery is not typically expected in patients in the chronic phase after stroke, the inclusion of a control group would have allowed a more precise quantification of intervention-related effects. Similarly, a longer follow-up period would be desirable to evaluate the long-term trajectory of functional outcomes and to determine whether, and after what time interval, patients might return toward baseline performance levels.

In addition to the limited sample size, a major limitation of the present study is the heterogeneity of the cohort with respect to stroke etiology (ischemic vs. hemorrhagic), time since stroke onset, and baseline functional levels across outcome measures. Emerging evidence suggests that baseline walking speed, for example, may substantially influence the magnitude of achievable improvement [31]. While recommending continuation of kinesiotherapy during the follow-up period is clinically appropriate, the absence of objective monitoring of adherence introduces additional uncertainty regarding the factors contributing to observed changes. The absence of immediate post-treatment assessment of ADL measures limits interpretation of short-term changes in functional independence. Furthermore, the lack of objective biomechanical or neurophysiological measures, such as gait kinematics or electromyography, limits insight into the underlying mechanisms of the observed functional improvements.

The retrospective design of the study is associated with a risk of selection bias. As the cohort was identified retrospectively from routine clinical records, patient flow could not be reconstructed in the same manner as in a prospective study. Only chronic post-stroke patients who completed the full rehabilitation program were included in the analysis, which may limit the representativeness of the sample.

Beyond addressing these limitations, future research could expand the range of assessed parameters and focus on the relationships between them. Examining how changes in gait kinematics relate to fear of falling, functional independence, walking speed, or cortical changes assessed by neuroimaging could provide valuable insight into mechanisms of recovery. In addition to neuromuscular stimulation techniques, adjunctive physical modalities such as radial extracorporeal shock wave therapy have been explored in post-stroke rehabilitation, particularly for the management of spasticity and motor dysfunction, and may represent a complementary approach within multimodal rehabilitation programs [44]. Longer follow-up periods may reveal meaningful patterns and help explain interindividual variability in treatment response, including why some patients demonstrate sustained improvement while others do not. Ultimately, such data could support the development of predictive or regression-based models to classify patients according to expected treatment response, thereby assisting clinicians in optimizing individualized rehabilitation protocols. Interindividual variability observed in the present study could support the future development of AI-driven or model-based FES systems that dynamically adjust stimulation parameters based on the patient’s current functional state, fatigue, or performance. Similar feedback-driven approaches have already been applied in robotic and AI-assisted upper-limb rehabilitation, where they have demonstrated increased effectiveness compared with conventional rehabilitation strategies [45]. Translating these concepts to lower-limb FES may allow more personalized therapy, improved precision of stimulation protocols, and a reduction in interindividual variability of treatment response.

Despite the pilot nature of the study and its associated limitations, the present findings may be considered a meaningful contribution to the field of rehabilitation in post-stroke patients. The results suggest a potential role for short-term facility-based multimodal rehabilitation incorporating FES in the management of patients in the chronic phase after stroke and indicate that functional changes may be maintained for several months following completion of treatment.

## 5. Conclusions

This retrospective pilot study suggests that a relatively short multimodal, facility-based rehabilitation program incorporating FES may be associated with improvements in gait mobility and independence in daily activities in patients with chronic post-stroke foot drop. Functional changes observed during the treatment course were maintained and, in some patients, further enhanced at the 3-month follow-up. Given the absence of a control group and the multimodal nature of the intervention, these findings cannot be attributed to FES alone and should be interpreted as exploratory and hypothesis-generating. Nevertheless, the results support the feasibility and potential clinical relevance of incorporating FES into short-term rehabilitation programs for chronic post-stroke patients. Further research is needed to confirm these results and to clarify the specific contribution of FES within comprehensive rehabilitation strategies.

## Figures and Tables

**Table 1:** Summary statistics of outcome measures at baseline, post-treatment, and follow-up.

		Mean ± SD	Median (IQR)
Barthel Index	Baseline	82.86 ± 17.6	87.5 (22.5)
	Post-treatment	88.21 ± 11.74	92.5 (17.5)
IADL	Baseline	5.93 ± 1.98	7 (2.5)
	Post-treatment	6.71 ± 1.22	7 (1.5)
10 MWT (m/s)	Baseline	0.78 ± 0.57	0.73 (1.1)
	Post-treatment	0.89 ± 0.6	0.81 (1.14)
	3-month FU	0.92 ± 0.61	0.72 (1.16)
TUG (s)	Baseline	25.12 ± 21.49	15.52 (29.62)
	Post-treatment	21.55 ± 20.57	13.26 (19.44)
	3-month FU	19.77 ± 19.36	13.09 (14.98)

FU: Follow-up; IQR: Interquartile range; SD: standard deviation.

**Table 2:** Results of the statistical analysis for individual outcome measures. Comparisons between time points were performed using the non-parametric Wilcoxon signed-rank test. A *p*-value < 0.05 was considered statistically significant.

		%Δ	*p* (<0.05)	Effect Size	MCID Responder Rate
Barthel Index	Baseline-Post	9.07 ± 13.49%	0.021	0.87	50%
IADL	Baseline-Post	23.64 ± 31.99%	0.019	0.88	64%
10 MWT (m/s)	Baseline-Post	19.05 ± 13.77%	<0.001	1.03	57%
	Baseline-FU	28.48 ± 30.45%	0.033	0.57	57%
TUG (s)	Baseline-Post	15.98 ± 9.05%	<0.001	1.03	50%
	Baseline-FU	19.10 ± 25.56%	0.01	0.68	57%

FU: Follow-up; MCID: minimal clinically important difference.

## Data Availability

The data presented in this study are available on request from the corresponding author. The data are not publicly available due to privacy and ethical restrictions.

## Abbreviations

The following abbreviations are used in this manuscript:

FES	Functional Electrical Stimulation
MCID	Minimal Clinically Important Difference
AFO	Ankle-foot orthosis
IADL	Instrumental Activities of Daily Living scale
10MWT	10-Meter Walk Test
TUG	Timed Up and Go
SD	Standard deviation
IQR	Interquartile range
AI	Artificial intelligence
